# Gut–Lung Axis: Microbial Crosstalk in Pediatric Respiratory Tract Infections

**DOI:** 10.3389/fimmu.2021.741233

**Published:** 2021-11-18

**Authors:** Wenxia Zhu, Yilin Wu, Hui Liu, Caini Jiang, Lili Huo

**Affiliations:** Shanghai Hospital of Integrated Traditional Chinese and Western Medicine, Shanghai University of Traditional Chinese Medicine, Shanghai, China

**Keywords:** gut microbiota, respiratory tract infection, pediatric, immune system, gut–lung axis

## Abstract

The gut microbiota is an important regulator for maintaining the organ microenvironment through effects on the gut-vital organs axis. Respiratory tract infections are one of the most widespread and harmful diseases, especially in the last 2 years. Many lines of evidence indicate that the gut microbiota and its metabolites can be considered in therapeutic strategies to effectively prevent and treat respiratory diseases. However, due to the different gut microbiota composition in children compared to adults and the dynamic development of the immature immune system, studies on the interaction between children’s intestinal flora and respiratory infections are still lacking. Here, we describe the changes in the gut microbiota of children with respiratory tract infections and explain the relationship between the microbiota of children with their immune function and disease development. In addition, we will provide perspectives on the direct manipulation of intestinal microbes to prevent or treat pediatric respiratory infections.

## Introduction

The intestinal tract is home to about 40 trillion microbiota, and the total number of its genes is about 150 times that of human genes. Given the size of the intestinal flora, it is also known as the “second genome”, “second brain”, or “gut brain” of the human body. Compared with the microbiota in other parts of the human body, the intestine contains huge bacterial groups and rich species. Therefore, it influences other organs and human body functions in diverse ways. It will soon be commonplace to treat various diseases with consideration of the intestinal microbiota.

## Intestinal Flora Involved in Systemic Immunity

**Figure 1:**
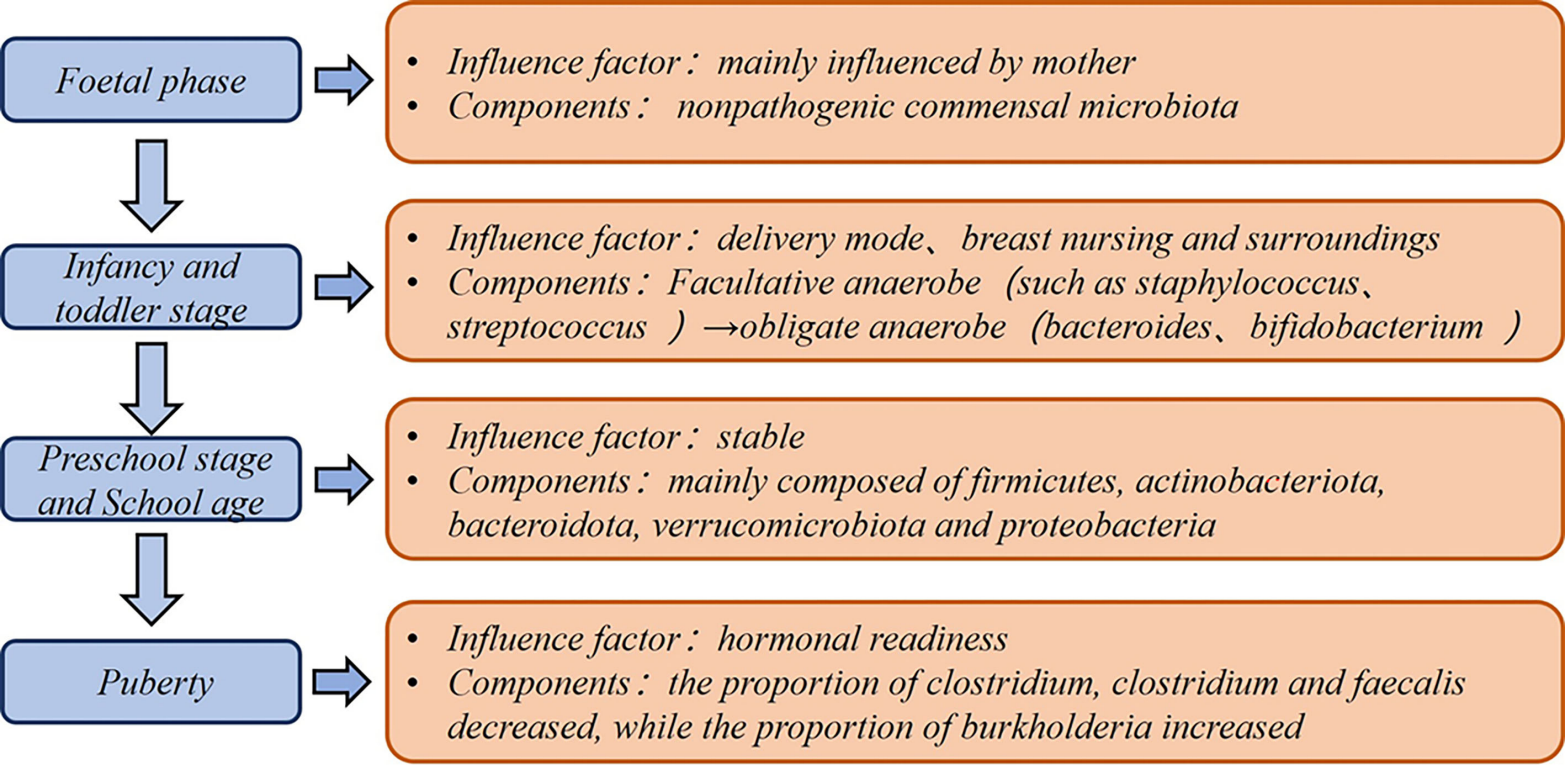
Dynamics of intestinal flora in children.

The gut microbiota is an increasingly recognized component of the human body that shows dynamic changes with age, region, diet, and medication (1) (**Figure 1**). Compared with other parts of the body, the intestinal microbiota possesses enormous bacterial taxa and a rich variety of species, and they are relatively easy to be used as targets for biological intervention. The roles of intestinal microbes include stimulating pattern recognition receptors (PRRs) as bacterial antigens, facilitating the maturation of host immune system (2), regulating the secretion of protective immunoglobulin A (IgA) in the intestine (3), affecting the synthesis and transport of neurotransmitters (4), and helping organisms synthesize vitamins and bile acids (5). These events obviously have profound impacts on the host’s immune response, nutrition metabolism, and cognitive function (6).

Despite the intestinal partition, the gut microbiota can promote inter-organ communication (5). This is achieved through microbe-produced metabolites. Short-chain fatty acids (SCFAs) are considered significant intestinal microbial metabolites, and acetic acid (C2), propionic acid (C3), and butyric acid (C4) account for approximately 95% of all SCFAs (7). SCFAs engage in cellular carbohydrate and fatty acid metabolism by regulating cell chromosome acetylation modification status through histone deacetylase inhibition (8, 9) or activating cells through the SCFA-G-protein receptor signal transduction pathway. These metabolites can promote immune cell maturation and maintain and regulate intestinal homeostasis *in situ*, but they also pass through intestinal tract into capillaries and transfer to major organs in the circulation. SCFAs have critical functions in the systemic action of the gut microbiota and play important roles in defense against infection, mitigation of autoimmune diseases, and anti-tumor therapy. In recent years, the description of the gut microbiota and its metabolites involved in respiratory diseases has gradually become more elaborate. With regard to the microbes themselves, microbial surface molecules like lipopolysaccharide (LPS) and lipoteichoic acids can be recognized by the immune system and induce corresponding humoral and cellular immune processes that can improve the host immune system. In controlled experiments on specific pathogen-free (SPM) mice and bacteria-free mice, SPF mice exhibited increased lymphatic tissue activity, more intestinal Peyer’s patches, and greater antibody secretion with less susceptibility to systemic inflammation (10–12). The mucosa is the body’s first line of defense against external pathogens.

The intestinal microbiota is essential for maturation of mucosal-associated immune tissues (MALT), which can induce the development of gut-associated immune tissues (GALT). Peyer’s patches, mesenteric lymph nodes, and isolated lymphoid follicles (ILFs) are important secondary lymphoid tissues of GALT and vital sites for the B cells that produce IgA to neutralize pathogenic microorganisms (13, 14). These structures are crucial habitats for IgA-producing plasma cells. In terms of their metabolites, SCFAs could ease microbial-induced allergic lung inflammation by acting on Th2 cells, while antibiotics such as vancomycin increase the incidence of allergic lung diseases by reducing intestinal SCFA levels (15). Moreover, SCFAs have the ability to alleviate pulmonary fibrosis and make patients less susceptible to further infection (16). C2–C4 can inhibit neutrophil cytotoxicity (17), and C3 has been reported to inhibit macrophage function in the intestine (18) by inducing the apoptosis of inflammatory dendritic cells (19), thereby affecting immunity in the whole body and all major organs. SCFA affects cells in various ways in different microenvironments. Various organs and microenvironments can induce the differentiation of initial T cells into effector T cells or T regulatory cells (9, 20, 21). Regarding the effect of SCFAs on B cells, one report indicated that it is associated with shaping intestinal homeostasis and maintaining IgA expression levels in the bronchial-associated mucosal immune system (22). Other recent findings suggest that SCFAs can protect against arthritis by skewing regulatory B cell differentiation (23).

## Dynamic Development of the Pediatric Intestinal Flora and Immune System

Although the fetus was previously considered to exist in a sterile uterine environment, the results of a 16S ribosomal DNA-based and whole-genome shotgun metagenomic study based of 320 subjects show that the placenta also has unique microbial niche that consists of nonpathogenic commensal microbiota (24). A recent study found that a small number of premature babies had placentas containing bacteria of the same origin as their mothers’ mouths (25). This suggests that even at the embryonic stage, the fetus is already in contact and exchanging information with microorganisms. In a retrospective analysis of newborn feces, an upward trend appeared in the variety and amount of intestinal microbes (26). The infant gut microbiota shows a rapid increase until 1 year of age. From 1 to 5 years of age, the growth rate of flora diversity decreases and the composition becomes more stable, but their gut microbiota is lower in both number and species compared to adults (27). HIT Chip microarray analysis indicated that the most striking differences between young children and adults are found in *Actinobacteria*, *Bacilli*, *Clostridium cluster IV*, and *Bacteroidetes* phylum-like groups (28). In terms of dietary habits including fat, protein, sugar, and fiber intake, nutritional intake in infants and older children can lead to dramatic differences in the colonized taxa (29). Before children reach puberty (7–12 years old), their gut microbiota exhibit different functions including vitamin synthesis, amino acid degradation, and oxidative phosphorylation (30).

Compared with adults, the shaping of the pediatric immune system is more dependent on education from the external environment. The gut microbiota provides a window into the immune maturation of newborns. After weaning from breast milk, the intestinal microflora expands and produces related SCFAs, giving the baby a mucosal immune stimulation and promoting immune system maturation (31, 32). Bacteria are necessary for ILF development. The addition of solid foods about 2 weeks after weaning can increase bacteria colonization and is thus conducive to enhancing infant gut microbiota integrity (13).

Some hypotheses suggest that children’s immune systems are not imperfect, or rather, offer them better protection at an early age. Compared to adults, children’s immune systems tend to be more protective to external invasions, but they also generate the corresponding immune response once activated (33). The pediatric immune system is in the process of development, from simple to complex and from tolerant to sensitive. Early adaptive immunity is not well developed, and relies more on the PRRs of innate immunity to recognize danger-related molecular patterns or pathogen-related molecular patterns (34, 35). Neonates have fewer neutrophils than adults, and the high level of interleukin (IL)-6 in infants will diminish neutrophil recruitment and thus inflammation (36, 37). Natural killer (NK) cells play a prominent role in controlling viral infections, but studies on human fetuses have shown that NK cells are extremely sensitive to transforming growth factor-β inhibition, thereby reducing cytotoxicity and interferon-γ production (38, 39). As for cellular and humoral immunity, infant T cell immunity skews towards Th2 cells, with reduced Th1 cell differentiation and immune responses (40). Due to less antigenic stimulation, neonates have fewer memory T cells and memory B cells than adults (41), and therefore are prone to mild responses following secondary stimulation. In a study of human volunteers of different ages (42), the proportions of CD4 RTE cells, transitional B cells, and CD8 RTE cells decreased with age; furthermore, the vitality of the thymus and bone marrow decreases. Several inflammatory cell subsets including Th1 cells, CD4IL-2 cells, CD8IL-2 cells, and invariant NK T cells are up-regulated with age. In contrast, Th2 and Th17 cells did not show age-related changes.

## Crosstalk Between Gut Microbiota and Childhood Respiratory Infections

In adult studies, the gut microbiota can directly regulate the immune function of the lungs (43). In an experiment to detect specific antibodies after influenza virus infection, neomycin-sensitive bacteria were shown to be related to the production of immune responses in the lungs (44). Dysbiosis of intestinal microbes can induce mice to have different Th cell responses to influenza virus infection. Th1 and Th2 cells are more controlled by the gut microbiota, with a weaker effect on Th17 cells (45). Follicular helper T cells are deficient in germ-free mice, leading to a lack of B cell humoral immunity and impaired IgA^+^ plasma cell function, which impacts the development and severity of colitis (46). Moreover, antibiotic use can dysregulate the intestinal microbe composition, which triggers the overgrowth of yeasts and more severe pulmonary allergic reactions (47). Common respiratory tract infections in children have been associated with the gut microbiota to some extent (48, 49). First of all, children are exposed to the outside world while their microbial colony gradually grows, and the immune system is constantly exposed to foreign antigens, which guide continuous immune system improvement. The gut microbiota shapes the immune system in children, providing constant reserves for the infectious diseases (50).

According to the results of 16S rRNA gene sequencing, *Lachnospira*, *Veillonella*, *Faecalibacterium*, and *Rothia* were reduced in the intestines of children with asthma, and fecal levels of SCFAs and intestinal-liver metabolites were also disordered. These four bacteria are associated with disease progression, and researchers proposed that low abundance of these bacteria contributes to a higher risk of developing asthma before the age of 3 (51). Respiratory syncytial virus (RSV) often causes severe lower respiratory tract infections in infants, but its pathogenesis remains obscure. Some studies have shown that the infant intestinal microbe niche is significantly related to RSV infection severity. The 16S rRNA gene sequencing results of the feces of infants infected with RSV showed higher abundance of S247, *Clostridiales*, *Odoribacteraceae*, *Lactobacillaceae*, and *Actinomyces* in moderately and severely infected patients compared to normal infants. The *Moraxellaceae* flora decreases in children with severe RSV (52). The correlation between the intestinal microbe niche and respiratory diseases in infants and young children is gradually being revealed for variety of diseases. A study of infant bronchitis (53) revealed that the four dominant bacterial groups in the intestinal tract of normal healthy infants were: *Escherichia*-dominant profile (30%), *Bifidobacterium*-dominant profile (21%), *Enterobacter/Veillonella*-dominant profile (22%), and *Bacteroides*-dominant profile (28%). However, the predominant taxa in children with bronchitis changed to: *Enterobacter/Veillonella*-dominant profile (15%), and *Bacteroides*-dominant profile (44%). Researchers hypothesized that the *Bacteroides*-dominant profile may put infants at a higher risk of developing bronchitis. This lays a theoretical foundation for modifying the gut microbiota to treat or prevent bronchitis. Lei Li and colleagues (54) reported reduced gut microbiota diversity in pediatric patients with recurrent respiratory tract infections. Conversely, patients were significantly enriched in *Firmicutes*, *Proteobacteria*, *Bacteroidetes*, *Actinobacteria*, *Verrucomicrobia*, *Tenericutes* phylas and *Enterococcus*, *Faecalibacterium*, *Bifidobacterium*, and *Eubacterium* generas. However, *Eubacterium*, *Faecalibacterium*, and *Bifidobacterium* decreased. Compared to their healthy peers, children with pulmonary tuberculosis had reduced intestinal microbe diversity with enrichment of the pro-inflammatory bacteria Prevotella and the opportunistic pathogen *Enterococcus*, and decreases in the probiotics *Ruminococcaceae*, *Bifidobacteriaceae*, and *Faecalibacterium prausnitzii* (55). It can be seen that there is a close relationship between intestinal flora and respiratory tract infection,different respiratory tract infection pathogens can cause different changes in intestinal flora (**Table 1**) .

**Table 1:** Changes in intestinal flora caused by respiratory pathogen infection.

Respiratory pathogens	Changes in intestinal flora	References
Respiratory syncytial virus	Firmicutes↓, S247↑, Clostridiales↑, Odoribacteraceae↑, Lactobacillaceae↑, Actinomyces↑	(52)
Influenza virus	Enterobacter↑, Akkermansia↓, Desulfovibrio↓, Lactobacillus↓	(56)
Mycoplasma	Bifidobacterium↓, Lactobacillus↓, Colibacillus↑	(57)
*Streptococcus pneumoniae*	Lactobacillus↓, Bifidobacterium↓, Bacteroidetes↓, Colibacillus↑	(58)
*Staphylococcus aureus*	Total aerobic↑, Enterococcus↑, Total anaerobic↓, *Clostridium perfringens*↑	(59)
Mycobacterium tuberculosis	Pro-inflammatory bacteria Prevotella↑, Opportunistic pathogen Enterococcus↑, Probiotics Ruminococcaceae↓, Bifidobacteriaceae↓, *Faecalibacterium prausnitzii*↓	(55)

The direction of the interaction between the gut microbiota and respiratory tract infections remains unclear. Whether fluctuations in the gut microbiota caused by environment, diet, or genetic factors increase the risk of respiratory tract infections, or whether respiratory tract infections skew the gut microbiota requires further investigation. What is certain is that intestinal microbial communities can indeed shape the pediatric immune system. In clinical treatment, altering the intestinal bacteria can indeed treat and prevent severe symptoms of respiratory diseases (60).

After cessation of breastfeeding, the intestinal microflora will expand and produce SCFAs, which will give the infant mucosal immune stimulation and promote immune system maturation (61). Some theories suggest that compared with adults, the pediatric immune landscape is indeed biased towards tolerance. For example, adults develop severe clinical symptoms of SARS-CoV-2 (COVID-19) infection, but only very few infected children develop dramatic upper respiratory symptoms (62). Children and adolescents often present with mild or no COVID-19 symptoms. In addition, young children have a very high probability of developing viral respiratory infections but with very low disease symptoms (63). This may be due to the fact that children are constantly undergoing gut microbiota remodeling and have a higher immune tolerance, which helps them resist the devastating effects of cytokine storms on the body.

## Gut Microbiota-Based Treatment for Respiratory Infections in Children

The gut microbiota provides protection against respiratory diseases by shaping the immune system. This role is even more pronounced in diseases for which innate immunity is involved in the early stage (64). For example, commensal bacteria transfer therapy can induce the production of neonatal intestinal innate lymphoid cells (ILCs), thereby boosting plasma granulocyte colony-stimulating factor levels and neutrophil numbers and improving IL-17-dependent sepsis tolerance (65). Resistance to respiratory infections can be significantly enhanced by using microbiome-related treatments rather than antibiotic therapies. LPS supplementation can increase the expression of IL-6 and IL-1, as well as the immune response to *E. coli*-induced pneumonia *via* Toll-like receptor 4 signaling (66). Germ-free mice secreted superfluous IL-10, creating an immunosuppressive microenvironment, which made them more susceptible to bacterial infection. Pretreatment with LPS can decrease IL-10 production and increase the infiltration of neutrophils into infected lungs (67). Intestinal segmented filamentous bacteria are critical in the formation of Th17 immunity against *S. aureus* pneumonia (68). The gut microbiota facilitates the maturation of IL-22^+^ILC3, offering host resistance to bacterial pneumonia (69). Microbial-based therapies are widely used in clinical trials in the form of probiotics. Compared with conventional antibiotics, intestinal microbiota transplantation can achieve better therapeutic outcomes (70, 71). Probiotics and supplements for infants and young children can alter the intestinal microbe composition (72), thereby reducing the risk of respiratory virus infection in premature infants. Supplementing *Lachnospira*, *Veillonella*, *Faecalibacterium*, and *Rothia* to mice with pneumonia significantly alleviated the symptoms, demonstrating the protective effect and therapeutic potential of specific gut microbiota taxa (73). The combination of vitamin C and probiotics (*Lactobacillus acidophilus CUL21*, *Lactobacillus acidophilus CUL60*, *Bifidobacterium bifidum CUL20*, and *Bifidobacterium animalis* subsp. lactis CUL34) can effectively lower the infection rate of the upper respiratory tract (74).

## Future Perspectives

The gut–lung axis theory is gradually being advanced and accepted. Stable development of the intestinal microbiota in children can improve resistance to pathogens that cause respiratory tract infections. As the organ with the largest microbial ecosystem, a healthy intestinal niche often shows rich diversity. During different respiratory diseases, microbes and products in the intestinal tract will turn into morbid types with harmful traits. In the disease state, there is more communication between the intestine and lungs. Mucosa, secretions, pH, and other changes act on target cells and become messengers between the gut and other organs.

In children, the immune system and gut microbiota are undergoing vigorous development. Given that the pediatric gut microbiota is undergoing an integrated developmental process, it is important for us to consider the potential impact of prematurely introducing or eliminating a certain bacteria. Shifts in gut microbiota early in life or before weaning may provoke an increase in asthma-related immune responses (49). Mild dietary interventions also make sense to treat respiratory infections in children. Given the development of the gut microbiota taxa, we should probably consider using antibiotics with caution. Microbiological intervention strategies should also be considered for children with respiratory infections with marked changes in the gut microbiota due to obesity, complicated enteritis, or immunodeficiency.

## Author Contributions

WZ was in charge of literature retrieval and paper writing. YW, HL, and CJ were in charge of literature retrieval. LH was in charge of reviewing the papers. All authors contributed to the article and approved the submitted version.

## Funding

Scientific Research Project of Shanghai Municipal Health Commission (202040407); Scientific Research Project of Shanghai Hongkou District Health Commission (HKQ-ZYY-2020-01).

## Conflict of Interest

The authors declare that the research was conducted in the absence of any commercial or financial relationships that could be construed as a potential conflict of interest.

## Publisher’s Note

All claims expressed in this article are solely those of the authors and do not necessarily represent those of their affiliated organizations, or those of the publisher, the editors and the reviewers. Any product that may be evaluated in this article, or claim that may be made by its manufacturer, is not guaranteed or endorsed by the publisher.
